# Neuronal and Endothelial Nitric Oxide Synthases in the Paraventricular Nucleus Modulate Sympathetic Overdrive in Insulin-Resistant Rats

**DOI:** 10.1371/journal.pone.0140762

**Published:** 2015-10-20

**Authors:** Qing-Bo Lu, Xue-Mei Feng, Ning Tong, Hai-Jian Sun, Lei Ding, Yu-Jiao Wang, Xuan Wang, Ye-Bo Zhou

**Affiliations:** 1 Key Laboratory of Cardiovascular Disease and Molecular Intervention, Department of Physiology, Nanjing Medical University, Nanjing 210029, China; 2 Clinical Laboratory of Luyi People's Hospital, Zhoukou 466000, China; 3 Neurology Department of Heze Municipal Hospital, Heze 274000, China; 4 Department of Pediatrics, the Fourth Clinical Medical College of Nanjing Medical University, Nanjing 210029, China; Universidade de São Paulo, BRAZIL

## Abstract

A central mechanism participates in sympathetic overdrive during insulin resistance (IR). Nitric oxide synthase (NOS) and nitric oxide (NO) modulate sympathetic nerve activity (SNA) in the paraventricular nucleus (PVN), which influences the autonomic regulation of cardiovascular responses. The aim of this study was to explore whether the NO system in the PVN is involved in the modulation of SNA in fructose-induced IR rats. Control rats received ordinary drinking water, whereas IR rats received 12.5% fructose-containing drinking water for 12 wks to induce IR. Basal SNA was assessed based on the changes in renal sympathetic nerve activity (RSNA) and mean arterial pressure (MAP) in response to chemicals administered to the PVN. We found an increased plasma norepinephrine level but significantly reduced NO content and neuronal NOS (nNOS) and endothelial NOS (eNOS) protein expression levels in the PVN of IR rats compared to Control rats. No difference in inducible NOS (iNOS) protein expression was observed between the two groups. In anesthetized rats, the microinjection of sodium nitroprusside (SNP), an NO donor, or Nω-nitro-L-arginine methyl ester (L-NAME), a non-selective inhibitor of NOS, into the PVN significantly decreased and increased basal SNA, respectively, in both normal and IR rats, but these responses to SNP and L-NAME in IR rats were smaller than those in normal rats. The administration of selective inhibitors of nNOS or eNOS, but not iNOS, to the PVN significantly increased basal SNA in both groups, but these responses were also smaller in IR rats. Moreover, IR rats exhibited reduced nNOS and eNOS activity in the PVN. In conclusion, these data indicate that the decreased protein expression and activity levels of nNOS and eNOS in the PVN lead to a reduction in the NO content in the PVN, thereby contributing to a subsequent enhancement in sympathoexcitation during IR.

## Introduction

Sympathetic abnormalities play an important role in the pathophysiology of cardiovascular disease associated with metabolic syndrome, diabetes mellitus and obesity [1–4]. In these diseases, insulin resistance (IR) is a common feature [5–8], and elevated sympathetic nerve activity (SNA) has been reported to be associated with IR [9,10]. Chronic and sustained sympathetic overdrive results in hypertension and the development of IR [11–13]. Increasing evidence indicates that central mechanisms are a major cause of the sympathetic enhancement observed during IR, obesity, diabetes and hypertension [9–13], such as an increase in excitatory transmitter, angiotension II and superoxide, but a decrease in inhibitory transmitter and NO, which would result in over-activationof the sympathetic nervous system. However, these complex mechanisms have not been completely elucidated with respect to IR.

The paraventricular nucleus (PVN) regulates sympathetic outflow and cardiovascular function under physiological or disease conditions such as hypertension, heart failure (HF), obesity, obesity-related hypertension and diabetes [14–18]. It has been reported that the PVN is one of the primary sites containing nitric oxide (NO)-positive neurons, and functional studies have demonstrated that NO in the PVN exerts inhibitory effects on SNA and participates in the modulation of cardiovascular activities [19]. A reduced content of the inhibitory neuromodulator NO has been suggested to cause the centrally mediated sympathetic overdrive observed in HF and hypertension [20,21]. The overexpression of neuronal NO synthase (nNOS) in the PVN alleviated the enhancement in renal SNA (RSNA) in HF model rats [20]. A previous study demonstrated that the nNOS level is decreased in HF, particularly in neurons of the PVN of the hypothalamus [22]. The inhibition of NO synthase (NOS) using the non-selective inhibitor Nω-nitro-L-arginine methyl ester (L-NAME) via microinjection into the PVN or intracerebroventricular injection elevated basal RSNA [23–26]. Furthermore, we found that both nNOS and endothelial NOS (eNOS) in the PVN were involved in the modulation of sympathetic overdrive in renovascular hypertensive rats [21]. It has been reported that nNOS dysfunction in the PVN participates in the progression of hypertension, HF and diabetes [21,27,28], and abnormal eNOS activity in the periphery or the PVN has also been implicated disease progression [29–31]. Moreover, inducible NOS (iNOS) in the PVN is involved in sympathoexcitation caused by restraint stress or corticotropin-releasing factor application [32,33]. Taken together, NO in the PVN is an important factor involved in the regulation of SNA not only in healthy animals but also in certain animal disease models [20,21,29]. However, whether the alteration of the NOS system in the PVN mediates the elevation in sympathetic outflow in the IR state has not been investigated.

The aim of this study was to investigate the role of NO and the NOS system (nNOS, eNOS and iNOS) in the PVN in sympathetic activation during IR. Our study was designed to explore the following: 1) the nitrite/nitrate (NOx) concentration (an index of NO) and the protein expression of nNOS, eNOS and iNOS in the PVN in Control and IR rats; 2) the effect of elevating the NO level in the PVN using the NO donor sodium nitroprusside (SNP) on basal SNA, as well as the basal SNA response to decreasing endogenous NO content via inhibition of NOS activity in the PVN using the non-selective NOS inhibitor L-NAME, in Control and IR rats; 3) the effect of the individual pharmacological inhibition of nNOS, eNOS and iNOS on basal SNA in Control and IR rats; and 4) the activity of nNOS and eNOS in the PVN in Control and IR rats.

## Materials and Methods

### Animals

Animals were randomly divided into 2 experimental groups: the Control group and the insulin-resistant (IR) group. All procedures followed the Guide for the Care and Use of Laboratory Animals (8th Edition, 2011), and the experimental protocols were approved by the Experimental Animal Care and Use Committee of Nanjing Medical University. Five-week old male Sprague–Dawley rats weighing 130–150 g were used for this experiment. The rats were housed in clear cages and were allowed free access to normal rodent diet and tap water under standard and constant conditions (12 h light/dark cycle and a controlled humidity and temperature of 22°C–26°C). The Control group received tap water for 12 wks, whereas IR was induced in the IR group by adding fructose (12.5%, AMRESCO, Solon, OH, USA) to the standard drinking water for 12 wks. IR is evidenced by an increase in both insulin and glucose levels, and it can be evaluated by HOMA-IR (the homeostasis model assessment of insulin resistance), namely IR index. HOMA-IR was calculated using the fasting plasma glucose and insulin levels according to the equation: HOMA-IR = glucose(mmol/L) × insulin (*μ*U/L)/22.5.

### Systolic blood pressure (SBP) measurement

From the beginning of the 11^th^ week to the end of the 12^th^ week, SBP was measured in each conscious rat using a non-invasive computerized tail-cuff system (NIBP; ADI, Bella Vista, NSW, Australia) as previously reported [21]. Each rat was monitored daily via an SBP measurement of the tail artery for at least 10 days before the initiation of the IR or Control model to minimize stress-induced fluctuations in SBP [34].

### Blood and PVN sample preparation

Briefly, blood was withdrawn from the tail tip of non-fasting rats for NE examination and fasting rats for detecting the levels of glucose, insulin and triglycerides using heparinized tubes, then plasma was obtained after centrifuging the blood at low speed centrifugation (3000 x g, 15 min) and stored at -80°C for biochemical analysis. The brain of the rat was rapidly removed, frozen in liquid nitrogen, and stored in a -80°C refrigerator [34]. Coronal sections of the brain were sliced using a cryostat microtome (CM1900, Wetzlar, Hessen, Germany) while referring to the rat brain atlas (Paxinos G and Watson C, 2005). Briefly, the region from 1.5 mm to 2.0 mm caudal to Bregma was considered as the region containing the PVN, and the brain tissue was sliced into a 450 μm coronal section that included the PVN area [34]. The PVN region was removed using a 15-gauge needle (inner diameter 1.5 mm) for all further measurements [35].

### Evaluation of basal sympathetic activity

For the evaluation of basal SNA, the plasma norepinephrine (NE) level was determined as an indirect index of sympathetic activity. The plasma NE level was examined using commercial ELISA kits (R&D Systems, Minneapolis, MN, USA) according to the manufacturer’s instructions as previously reported [17]. Briefly, the 96-well plates were incubated in an antibody specific for rat NE. The samples and standard diluent buffer were applied to the 96-well plates, incubated and washed. Then, a horseradish peroxidase-conjugated secondary antibody solution was administered, and the reaction was terminated using stop solution. The final solution was read at a λ of 450 nm using a microplate reader (ELX800, BioTek., Winooski, VT,USA) [17].

### Measurement of the plasma glucose, insulin and triglyceride levels

Blood was collected from the tail tip of Control and IR rats at the end of 12 wks. Using the glucose oxidase method, fasting plasma glucose was measured using a kit (Jiancheng Bioengineering, Nanjing, Jiangsu, China); the fasting plasma insulin level was determined via enzyme-linked immunosorbent assay (ELISA) using a kit (RayBiotech, Inc., Norcross, GA, USA); the plasma triglyceride level was detected via a colorimetric assay using a commercial kit (Jiancheng Bioengineering, Nanjing, Jiangsu, China). The manufacturer’s instructions were followed for each measurement process [35].

### Measurement of the NO metabolite (NOx) levels in the PVN

The nitric oxide metabolite (NOx) content is widely used as an index of the NO level. NO production in the PVN was evaluated based on the detection of the concentration of its stable metabolites nitrate and nitrite using a Nitrate/Nitrite Colorimetric Assay Kit (Cayman Chemical Co., Ann Arbor, MI, USA) [21]. Total protein was extracted from the PVN homogenate according to the manufacturer’s instructions [21]. The results are expressed as nmol/mg of protein.

### Measurement of eNOS, nNOS and iNOS protein expression

Western blot was used to determine the nNOS, eNOS and iNOS protein expression levels. The protein expression levels of eNOS, nNOS and iNOS in the PVN were measured as previously reported [21]. Briefly, PVN tissue samples were collected and homogenized. The concentration of total protein that was extracted from PVN homogenates, was determined by the method described by Bradford [36] using a total protein quantitative assay kit from Nanjing Jiancheng Biotechnology Co. (A045-2, Nanjing, Jiangsu, China). Western blot analysis was performed using rabbit polyclonal antibodies against eNOS (1:2000, Cell Signaling Technology, Danvers, MA, USA), nNOS (1:2000, Cell Signaling Technology, Danvers, MA, USA), iNOS (1:1000, Santa Cruz, CA, USA) and GAPDH (1:2000, Bioworld Technology, Louis Park, MN, USA) as the primary antibodies. The secondary antibody was peroxidase-conjugated goat anti-rabbit IgG (Santa Cruz, CA, USA). GAPDH was used as a loading control, and the protein expression levels of eNOS, nNOS and iNOS were normalized to the GAPDH protein level.

### Assessment of the dimer-monomer ratio of nNOS and eNOS in the PVN

To quantify active nNOS and eNOS dimers and inactive monomers (nNOS and eNOS) in the PVN, we performed low-temperature polyacrylamide gel electrophoresis (LT-PAGE) as described previously [28,37]. Before fractionation in a 5% separating gel, standard Laemmli buffer was applied to 60 μg of PVN protein at 4°C for 30 min. For the analysis of dimeric and monomeric nNOS and eNOS, the samples were not heated, and the buffers and gels used were pre-equilibrated to the same temperature (4°C). Both the electrophoresis and transfer processes were performed on ice.

### General procedures of the acute experiment

At the end of the 12th week, each rat was anesthetized via intraperitoneal injection of urethane (800 mg/kg) and α-chloralose (40 mg/kg). A supplemental dose of anesthesia was needed to maintain a suitable level of anesthesia during the experiment [34,35]. The rat was ventilated with room air using a rodent ventilator (model 683, Harvard Apparatus Co. Inc., South Natick, MA, USA). To continuously record arterial blood pressure (ABP) and mean arterial pressure (MAP), the right carotid artery of the rat was cannulated and connected to a pressure transducer (MLT0380, AD Instruments, Bella Vista, NSW, Australia) [34,35].

### RSNA recordings

The left renal sympathetic nerve was isolated via a retroperitoneal incision. The renal nerve was cut distally to eliminate afferent activity and was placed on a pair of silver electrodes [34,35]. To amplify the nerve signals, a four-channel AC/DC differential amplifier (DP-304, Warner Instruments, Hamden, CT, USA) was used. The data were collected using a high pass filter at 10 Hz and a low pass filter at 3,000 Hz, and RSNA was integrated at a time constant of 100 ms [34,35]. At the end of each experiment, background noise was detected after section of the central end of the renal nerve at the end of the experiment and was subtracted from the integrated values of the recorded RSNA [21,34,35]. The change in RSNA was expressed as the percent change from baseline [21]. Baseline RSNA and MAP were determined by averaging 2 min of the maximal RSNA responses after microinjection into the PVN.

### PVN microinjection

**Fig 1:**
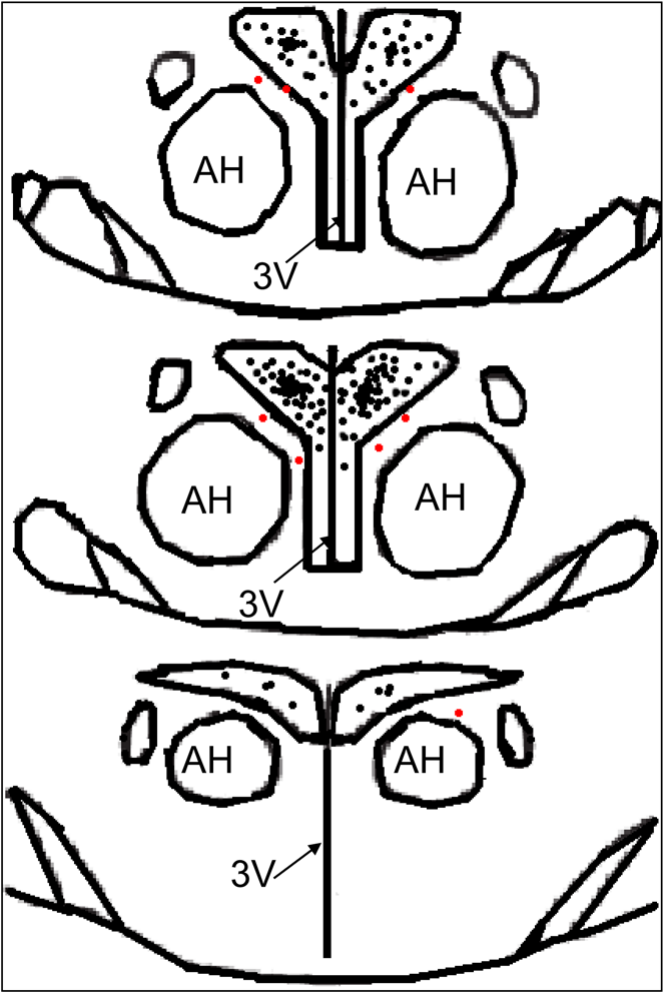
A schematic representation of the microinjection sites in the PVN region from the rostral (1.6) to the caudal (2.1) extent. The black or red dots represent the termination sites of microinjection into the PVN. The black dots were considered to be within the PVN, and the red dots were considered to be at the margin of the PVN or outside the PVN; the rats injected at such sites were excluded from data analysis. The distance, in millimeters, posterior to Bregma is shown in each section according to the Paxinos & Watson rat atlas. AH, anterior hypothalamic nucleus; PVN: paraventricular nucleus; 3V: third ventricle.

The stereotaxic coordinates of the PVN location were set as 1.8 mm caudal to Bregma, 0.4 mm lateral to the midline and 7.9 mm ventral to the dorsal surface of the brain according to the Paxinos & Watson rat atlas [21]. The microinjection volume was 50 nL for each side of the PVN, and the bilateral PVN microinjections were completed within 1 min [34]. At the end of the experiment, 2% Evans Blue dye (50 nL) was injected into each site to identify the PVN injection sites under a microscope [34]. If the microinjection site was outside the PVN or was at the margin of the PVN, the rat was excluded from data analysis [35]. A schematic representation of the microinjection sites in the PVN region is shown in **Fig 1**.

### Chemicals

L-NAME, SNP and the selective iNOS inhibitor S-methylisothioureahemisulfate salt (SMT) were obtained from Sigma Chemical (St. Louis, MO, USA). The selective nNOS inhibitor N-propyl-L-arginine (N-Propyl) and the eNOS inhibitor N5-(1-iminoethyl)-L-ornithine (L-NIO) were obtained from Tocris Bioscience (Bristol, UK). These chemicals were dissolved in normal saline. The doses of L-NAME (50, 100 nmol), SNP (25, 50 nmol), SMT (250, 500 pmol), N-Propyl (0.5 nmol, 5 nmol) and L-NIO (5 nmol, 50 nmol) applied in this experiment were selected based on our preliminary studies and published reports [21,28,32].

### Protocols

#### Experiment 1

Blood samples were collected to measure the plasma NE levels, and the brains were removed to determine the nNOS, eNOS and iNOS protein expression levels in the PVN in a subset of Control rats and a subset of IR rats (n = 8 for each subset: 4 rats from each group for the measurement of nNOS and eNOS protein expression in the PVN, and the remaining 4 rats from each group for the detection of the iNOS protein levels in the PVN).

#### Experiment 2

The effects of the microinjection of saline, L-NAME (50, 100 nmol), SNP (25, 50 nmol), N-Propyl (0.5 nmol, 5 nmol), L-NIO (5 nmol, 50 nmol) and SMT (250, 500 pmol) into the PVN on baseline RSNA and MAP were determined in the Control and IR rats (n = 6 for each dosage.). Each rat only received two injections on either side of the PVN in this experiment. The interval between the two injections was approximately 2 hrs, and the microinjection volume for each side of the PVN was 50 nL for each chemical [35].

#### Experiment 3

The brains were removed to determine the NOx levels (n = 6 for each group) and the dimer-monomer ratios of nNOS and eNOS (n = 4 for each group) in the PVN in the Control and IR rats.

### Statistical analysis

According to the experimental design, we needed at least 84 Control rats and 84 IR rats in the experiment. Finally, a few of the rats were excluded due to surgical failure, failure to meet the criteria for IR after fructose feeding in IR group based on homoeostasis model assessment of insulin resistance (HOMA-IR), or misplacement of the microinjection sites relative to the PVN location [34,35]. A total of 92 normally fed rats and 123 fructose-fed rats (to induce IR) were used to meet the requirements for this experiment. Among the Control rats, a total of 8 rats were excluded due to surgical failure of the acute experiment (4 rats) or missing the PVN (4 rats). Additionally, 39 IR rats were excluded; among them, 32 rats did not meet the criteria for IR, 4 rats were not microinjected at the PVN location, and 3 rats experienced surgical failure of the acute experiment performed 12 wks after fructose feeding. Experimental data were successfully obtained from 84 Control rats and 84 IR rats that met the criteria for IR following fructose feeding in this experiment. For all rats in the IR group, the glucose, insulin and triglycerides levels were measured (at the end of the 12th week; the rats were fasted overnight but were provided with free access to water, and the blood from tail tip was used to examine the levels of glucose, insulin and triglycerides) [34,35]. All data were expressed as the means±SE. Student's t-test was used for comparisons between the control and IR rats. One-way ANOVA followed by the Bonferroni test for post hoc analysis was used for multiple comparisons. P<0.05 was considered to be statistically significant.

## Results

### Metabolic data

It has been reported that the fructose-fed rat model develops hyperinsulinemia, IR, hypertriglyceridemia, and mild hypertension [38]. The analyses of the effect of fructose feeding on the plasma glucose, insulin, and triglyceride levels and HOMA-IR in the two groups are shown in **Table 1**. At the end of the 12th week, the HOMA-IR was significantly increased in the IR group compared with the Control group (P<0.05). The rats in the IR group met the HOMA-IR threshold for IR [6.78±0.47 (IR) vs. 2.48±0.19 (Control), p<0.05]. For the present study, experiments were performed on IR rats that met the criteria for IR based on the HOMA-IR. The plasma glucose, insulin and triglyceride levels also significantly elevated in the IR group [glucose: 6.21±0.49(mmol/l), insulin: 24.57±2.95 (μU/mL) and triglyceride: 131.7±9.12 (mg/dl)] compared with Control rats [glucose: 4.99±0.21 (mmol/l), insulin: 11.20±1.23 (μU/mL) and triglyceride: 52.02±3.45 (mg/dl); P<0.05 for each, **Table 1**].

**Table 1:** Metabolic characteristics of the Control and IR rats at the end of the 12th week.

	Control	IR
**Plasma glucose (mmol/l)**	4.99±0.21	6.21±0.49*
**Plasma insulin (**μ**U/ml)**	11.20±1.23	24.57±2.95*
**HOMA-IR**	2.48±0.19	6.78±0.47*
**Plasma triglyceride (mg/dl)**	52.02±3.45	131.7±9.12*

The values are presented as the means±SE.

*P<0.05 compared with the Control group.

n = 6 for each group. IR: insulin resistance; HOMA-IR: homoeostasis model assessment of insulin resistance. HOMA-IR = Fasting plasma insulin (μU/ml) x Fasting plasma glucose (mmol/l)/22.5.

### General anatomical and haemodynamic characteristics

Hypertension can be induced by feeding a fructose-rich diet, demonstrating a link between fructose ingestion and increased blood pressure (BP) [38]. In this study, the rats that consumed fructose for 12 wks gradually developed mild hypertension (SBP: 142±8 mmHg) compared to the Control rats (109±5 mmHg) (p<0.05). This hypertension was accompanied by an elevation in MAP in the IR rats [123±9 (IR) vs. 97±6 mmHg (Control), p<0.05]. Heart weight and the heart-to-body weight ratio, but not the body weight or the heart rate, of the fructose-fed rats were significantly increased compared to the normally fed rats, as shown in **Table 2**. These data agree with the characteristics of fructose intake in rats [34,35,39,40].

**Table 2:** Anatomic and hemodynamic characteristics of the Control and IR rats at the end of the 12th week.

	Control	IR
**BW (g)**	515±26	532±29
**HW (mg)**	1201±43	1679±71*
**HW/BW (mg/g)**	2.33±0.09	3.15±0.20*
**Heart rate (beats/min)**	345±21	364±25
**SBP (mmHg)**	109±5	142±8*
**MAP (mmHg)**	97±6	123±9*

IR: insulin resistance; HW: heart weight; BW: body weight; SBP: systolic blood pressure; MAP: mean arterial pressure. n = 6 for each group. The values are presented as the means±SE.

*P<0.05 compared with the Control group.

SBP was measured in the conscious state, and MAP and HR were determined under anesthesia.

### The levels of NE in plasma and of NOx in the PVN

**Fig 2:**
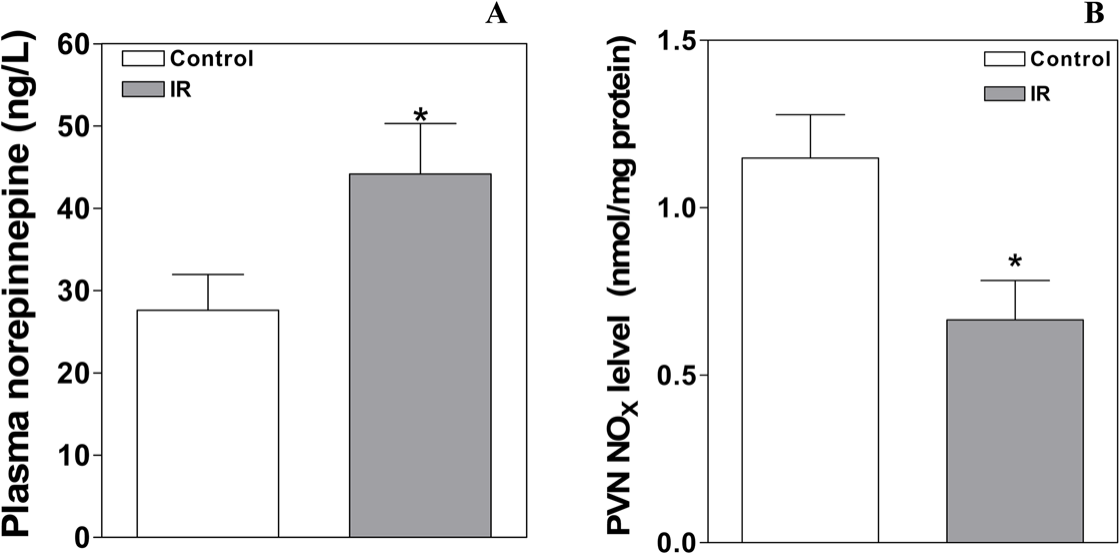
The plasma NE and NOx levels in the PVN at the end of the 12th week in the Control and IR rats. The values are presented as the means±SE; n = 8 for the plasma NE level and n = 6 for the PVN NOx level for each group. *P<0.05 vs. the Control group. PVN: paraventricular nucleus; IR: insulin resistance.

The plasma NE level (**Fig 1A**), which was used to evaluate basal SNA, was increased in the IR group compared to the Control group [45.2±5.7 (IR) vs. 27.6±4.3 (Control) ng/L, p<0.05, **Fig 2A**]. The NOx level is widely used as an index of the NO level [21]. The level of NOx in the PVN in the Control and IR rats is presented in **Fig 2B**. The basal NOx content in the PVN was significantly reduced in IR rats compared with Control rats (0.67±0.12 vs. 1.15±0.13 nmol/mg protein, P<0.05).

### The levels of nNOS, eNOS and iNOS protein expression in the PVN

**Fig 3:**
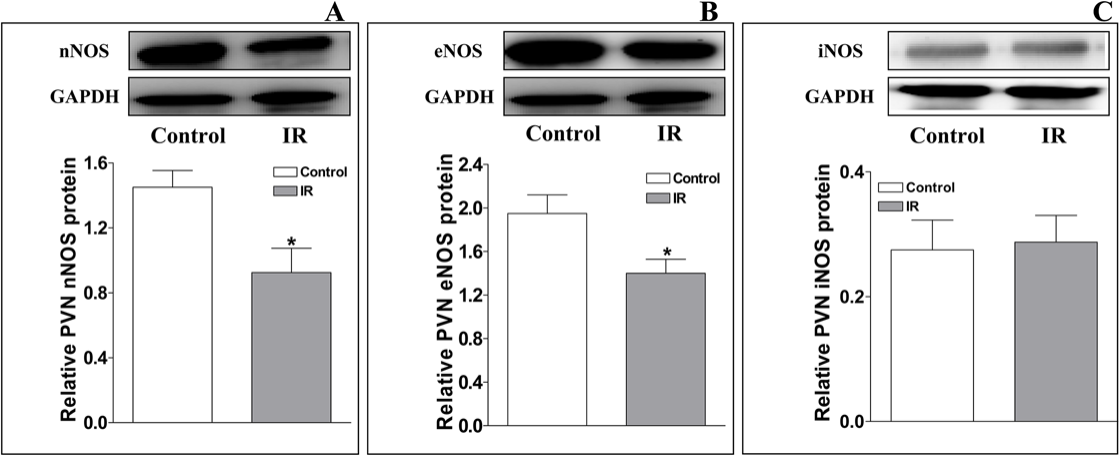
Relative levels of nNOS, eNOS, and iNOS protein expression in the PVN at the end of the 12th week in the Control and IR rats. The values are presented as the means±SE. *P<0.05 vs. the Control group. n = 4 for each group. PVN: paraventricular nucleus; IR: insulin resistance.

Representative Western blot analysis of total endogenous nNOS, eNOS and iNOS protein expression in the PVN from Control and IR rats is shown in **Fig 3**. Compared with the Control group, the levels of nNOS and eNOS protein expression in the PVN were significantly decreased in the IR group [relative to GAPDH protein expression; nNOS: 0.93±0.15 (IR) vs. 1.45±0.10 (Control); eNOS: 1.40±0.13 (IR) vs. 1.95±0.17 (Control); P<0.05 for each, **Fig 3A and 3B**], but the iNOS protein expression level was not significantly different between the two groups [iNOS: 0.29±0.04 (IR) vs. 0.28±0.05 (Control), P>0.05, **Fig 3C**].

### Effects of L-NAME and SNP on basal SNA in the PVN and MAP

**Fig 4:**
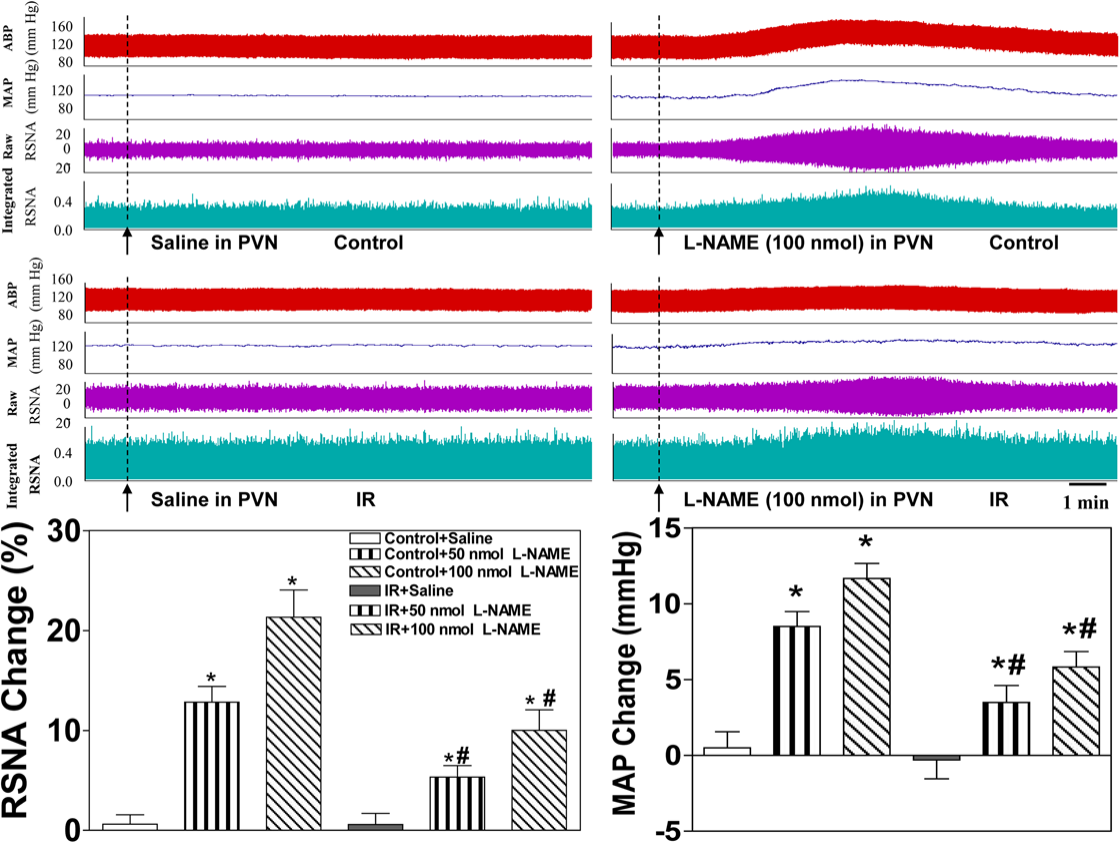
Effects of the microinjection of saline or the non-selective NOS inhibitor L-NAME (50 nmol, 100 nmol) into the PVN on baseline RSNA and MAP in the Control and IR rats. Upper panel: Representative traces demonstrating the effects of saline and L-NAME injection into the PVN on baseline RSNA and MAP in the Control and IR rats. Lower panel: Percent change in RSNA and MAP after the administration of saline or L-NAME to the PVN of the Control and IR rats. The values are presented as the means±SE. *P<0.05 vs. saline. ^#^P<0.05 vs. the Control group. n = 6 for each dosage. Each rat only received two injections namely saline and L-NAME infusion on either side of the PVN, and 18 Control rats and 18 IR rats were used in this experiment. PVN: paraventricular nucleus; RSNA: renal sympathetic nerve activity; MAP: mean arterial pressure.

**Fig 5:**
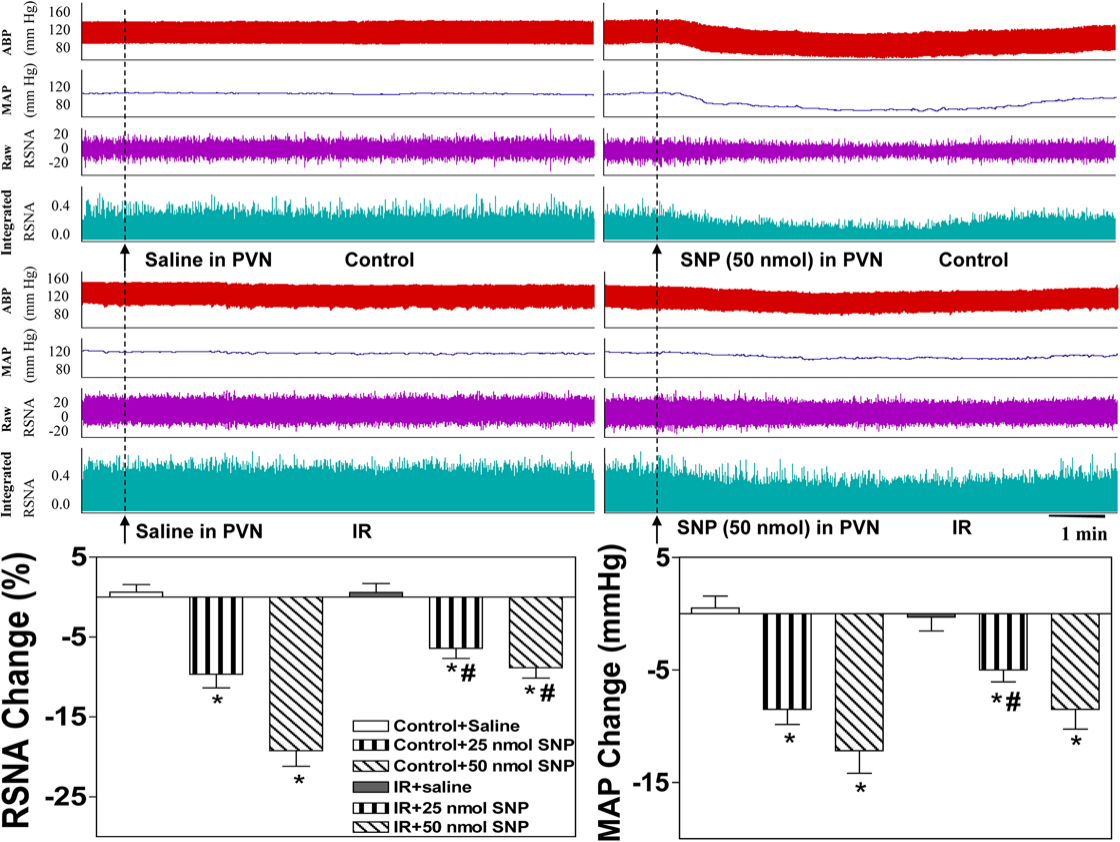
Effects of the microinjection of saline or the NO donor SNP (25 nmol, 50 nmol) into the PVN on baseline RSNA and MAP in the Control and IR rats. Upper panel: Representative traces demonstrating the effects of saline and SNP injection into the PVN on baseline RSNA and MAP in the Control and IR rats. Lower panel: Percent change in RSNA and MAP after the administration of saline or SNP to the PVN in the Control and IR rats. The values are presented as the means±SE. *P<0.05 vs. saline. ^#^P<0.05 vs. the Control group. n = 6 for each dosage. Each rat only received two injections namely saline and SNP infusion on either side of the PVN, and 12 Control rats and 12 IR rats were used in this experiment. PVN: paraventricular nucleus; RSNA: renal sympathetic nerve activity; MAP: mean arterial pressure.

The peak pressor and sympathoexcitatory responses to the microinjection of L-NAME into the PVN occurred approximately 5–15 min after the injection, and the peak depressor and sympathoinhibitory responses to the microinjection of SNP into the PVN occurred approximately 5–10 min after the injection. The microinjection of the non-selective NOS inhibitor L-NAME into the PVN significantly elevated baseline RSNA and MAP in the Control and IR rats **(Fig 4**, lower panel). The microinjection of the NO donor SNP into the PVN significantly lowered baseline RSNA and MAP in the Control and IR groups (**Fig 5**, lower panel). However, these responses to LAME and SNP in the IR rats were smaller than those in the Control rats. All values for the changes in baseline RSNA and MAP in response to saline and high-dose L-NAME and SNP in the Control and IR rats are shown in **Table 3**, and representative traces of the RSNA and MAP responses to LAME or SNP injection into the PVN in the Control and IR group are presented (**Figs 4 and 5**, upper panel).

**Table 3:** Changes in baseline RSNA (%) and MAP (mmHg) in response to the microinjection of saline, L-NAME (100 nmol), SNP (50 nmol), Nω-propyl (5 nmol), L-NIO (50 nmol) or SMT (500 pmol) into the PVN of the Control and IR rats.

	Control	IR
**Saline; L-NAME (ΔRSNA)**	0.62±0.94; 21.3±2.7*	0.58±1.11; 10.0±2.1* ^,^ ^#^
**Saline; L-NAME (ΔMAP)**	0.50±1.06; 11.7±1.0*	-0.31±1.24; 5.8±1.0*
**Saline; SNP (ΔRSNA)**	0.62±0.94; -19.2±1.9*	0.58±1.11; -8.9±1.3* ^,^ ^#^
**Saline; SNP (ΔMAP)**	0.50±1.06;–12.2±2.0*	-0.31±1.24; -8.5±1.8*
**Saline; Nω-propyl (ΔRSNA)**	0.62±0.94; 16.0±2.4*	0.58±1.11; 8.1±1.3* ^,^ ^#^
**Saline; Nω-propyl (ΔMAP)**	0.50±1.06; 9.3±1.5*	-0.31±1.24; 4.7±1.3* ^,^ ^#^
**Saline; L-NIO (ΔRSNA)**	0.62±0.94; 9.8±1.4*	0.58±1.11; 3.7±0.8* ^,^ ^#^
**Saline; L-NIO (ΔMAP)**	0.50±1.06; 4.8±0.9*	-0.31±1.24; 1.3±0.9^#^
**Saline; SMT (ΔRSNA)**	0.62±0.94; 0.95±1.03	0.58±1.11; 0.05±1.30
**Saline; SMT (ΔMAP)**	0.50±1.06; 0.36±1.31	-0.31±1.24; 0.13±1.00

The values are expressed as the means±SE. n = 6 for each group.

*P<0.05 vs. saline.

^#^P<0.05 vs. the Control group.

PVN: paraventricular nucleus; RSNA: renal sympathetic nerve activity; MAP: meanarterial pressure; IR: insulin resistance. For each side of the PVN, the microinjection volume was 50 nL for each chemical used in this study. Baseline RSNA and MAP were determined based on a 2-min average of their maximal response after PVN microinjection.

### Effects of injecting the selective nNOS inhibitor N-Propyl, the selective eNOS inhibitor L-NIO or the selective iNOS inhibitor SMT into the PVN on baseline SNA and MAP

**Fig 6:**
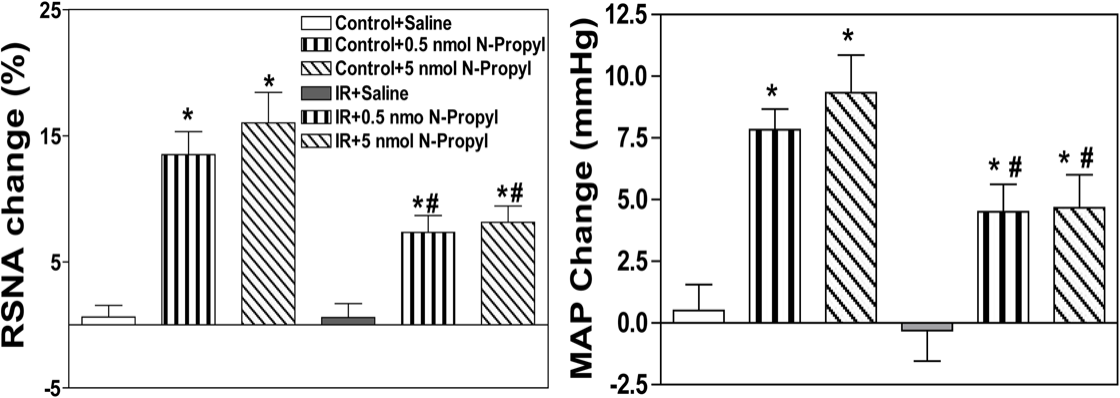
Effects of the microinjection of saline or the selective nNOS inhibitor N-Propyl (0.5 nmol, 5 nmol) into the PVN on baseline RSNA and MAP in the Control and IR rats. PVN: paraventricular nucleus; RSNA: renal sympathetic nerve activity; MAP: mean arterial pressure; IR: insulin resistance. n = 6 for each dosage. Each rat only received two injections namely saline and N-Propyl infusion on either side of the PVN, and 12 Control rats and 12 IR rats were used in this experiment. The values are presented as the means±SE. *P<0.05 vs. saline. ^#^P<0.05 vs. the Control group.

**Fig 7:**
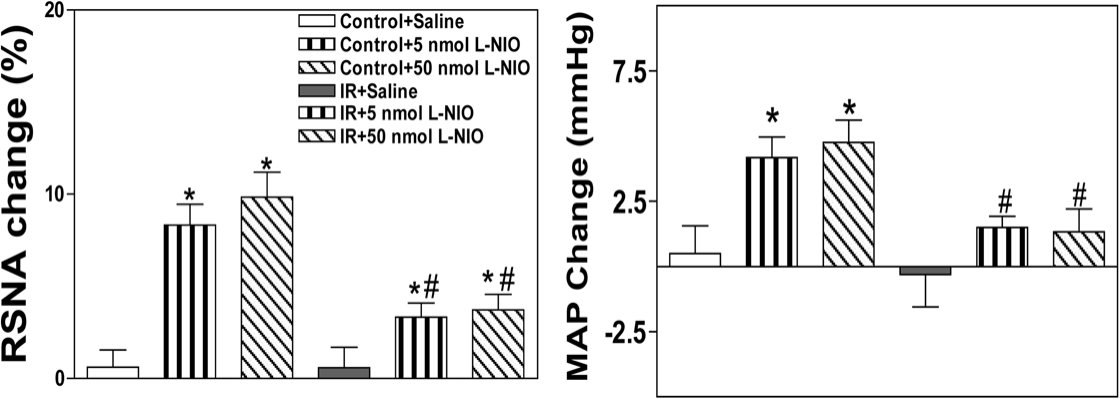
Effects of the microinjection of saline or the selective eNOS inhibitor L-NIO (5 nmol, 50 nmol) into the PVN on baseline RSNA and MAP in the Control and IR rats. PVN: paraventricular nucleus; RSNA: renal sympathetic nerve activity; MAP: mean arterial pressure; IR: insulin resistance. n = 6 for each dosage. Each rat only received two injections namely saline and L-NIO infusion on either side of the PVN, and 12 Control rats and 12 IR rats were used in this experiment. The values are presented as the means±SE. *P<0.05 vs. saline. ^#^P<0.05 vs. the Control group.

**Fig 8:**
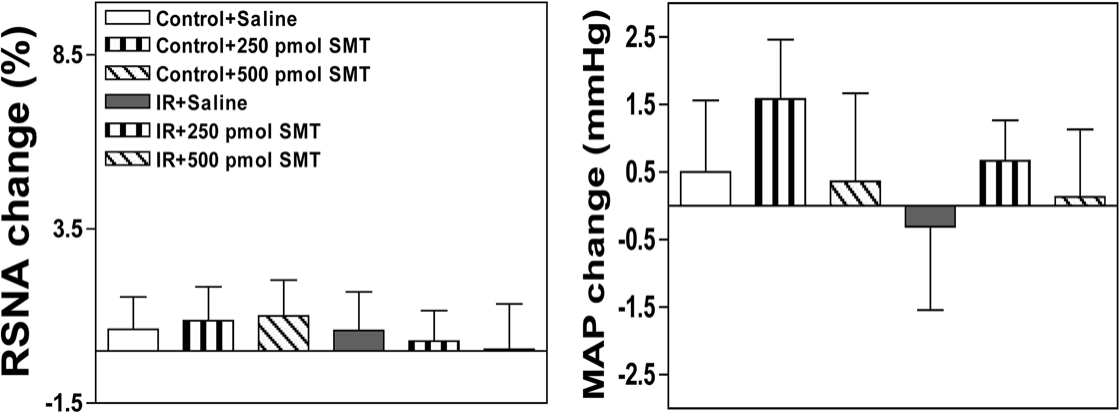
Effects of the microinjection of saline or the selective iNOS inhibitor SMT (250 pmol, 500 pmol) into the PVN on baseline RSNA and MAP in the Control and IR rats. PVN: paraventricular nucleus; RSNA: renal sympathetic nerve activity; MAP: mean arterial pressure; IR: insulin resistance. n = 6 for each dosage. Each rat only received two injections namely saline and SMT infusion on either side of the PVN, and 12 Control rats and 12 IR rats were used in this experiment. The values are presented as the means±SE.

The peak pressor and sympathoexcitatory responses to the microinjection of N-propyl or L-NIO into the PVN occurred approximately 5–10 min after the injection. The microinjection of either the selective nNOS inhibitor N-Propyl or the selective eNOS inhibitor L-NIO into the PVN markedly elevated baseline RSNA and MAP in both groups, but these responses were smaller in IR rats than in Control rats (**Figs 6 and 7**). However, no significant alteration in baseline RSNA or MAP was observed between the two groups following the microinjection of the selective iNOS inhibitor SMT into the PVN (**Fig 8**). All values for the baseline RSNA and MAP responses to high-dose N-propyl, L-NIO and SMT in the Control and IR rats are shown in **Table 3**. The microinjection of L-NAME, SNP, Nω-propyl, SNP, L-NIO or SMT outside the PVN did not change basal SNA (data not shown).

### The dimeric to monomeric nNOS and eNOS expression ratios in the PVN

**Fig 9:**
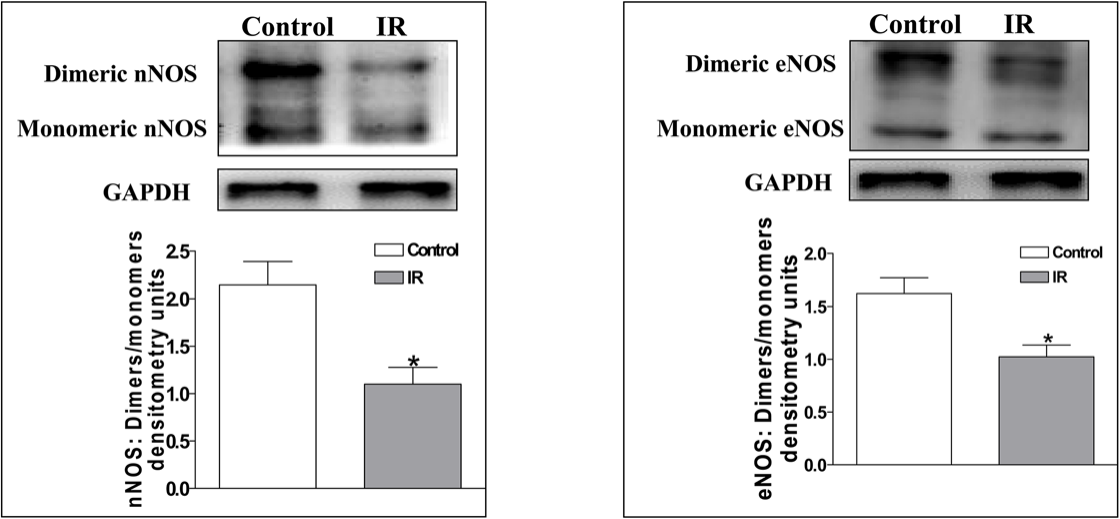
The dimeric/monomeric expression ratio of nNOS (A) and eNOS (B) in the PVN in the Control and IR rats. The values are presented as the means±SE. *P<0.05 vs. the Control group. n = 4 for each group. PVN: paraventricular nucleus; IR: insulin resistance.

The decrease in the NO level in the PVN of IR rats may be related to the reduced nNOS and eNOS activity levels in the PVN. Thus, we examined the expression ratios of dimeric to monomeric nNOS and eNOS, which reflect the nNOS and eNOS activity levels, in the PVN of Control and IR rats. The active homodimeric form of nNOS and eNOS is necessary for NO synthesis, and the activity levels of nNOS and eNOS can be determined by evaluating dimeric/monomeric expression ratio [36]. We found that the dimeric/monomeric expression ratios were significantly lower in IR rats than in Control rats (nNOS: 1.10±0.18 vs. 2.15±0.24; eNOS: 1.03±0.11 vs. 1.63±0.15; P<0.05 for each, **Fig 9**).

## Discussion

The findings in this study indicate that in IR rats, we observed 1) a decreased level of NO in the PVN that can be represented by the reduced NOx level (**Fig 2B**), 2) a reduced protein level of nNOS and eNOS in the PVN, 3) an increase in basal SNA in response to the microinjection of the non-selective NOS inhibitor L-NAME into the PVN, 4) a decrease in basal SNA in response to the microinjection of the NO donor SNP, and 5) an increase in RSNA and MAP in response to the microinjection of either the selective nNOS inhibitor N-Propylor the selective eNOS inhibitor L-NIO into the PVN. The basal SNA responses to L-NAME, SNP, N-Propyl and L-NIO were smaller in IR rats than in Control rats. Moreover, IR rats displayed a lower dimeric/monomeric expression ratio of nNOS and eNOS. These data suggest that endogenous nNOS and eNOS are involved in the modulation of SNA and that the decreased protein expression and activity levels of nNOS and eNOS may be a major cause of NO deficiency in the PVN, resulting in elevated SNA during IR.

NO not only acts as a neuronal messenger but also performs various physiological functions, including acting as a nonconventional neurotransmitter to modulate SNA [19,24,25,41–43]. Recent findings have suggested that endogenous NO in the PVN participates in the modulation of SNA based on the direct detection of RSNA [21,25,24]. For instance, decreased NO content in the PVN results in sympathetic activation during hypertension and diabetes [21,23,29]. Therefore, it can be speculated that the decreased or increased production of NO in the PVN promotes or inhibits sympathetic overdrive, respectively, during IR. In this study, there was a decreased NO content in the PVN in IR rats, but the plasma NE level was increased, and high dosage of L-NAME in the PVN did not cause stronger effect on SNA, which indicate that NO decrease in the PVN does not suppress the sympathetic overdrive sufficiently in IR. To elucidate the role of PVN NO in the regulation of SNA, we administered L-NAME or SNP to the PVN. Although L-NAME and SNP can cause a significant increase and decrease in SNA in IR rats, respectively, we found that their effects on IR rats were smaller than those on normal rats by comparison andthis effect was not strengthened by using larger dosage of L-NAME or SNP in the PVN. These blunted responses in IR rats are probably due to reduced NO or depressed neuronal response to NO that may be affected by oxidative stress or inflammation. However, the precise downstream mechanisms remain to be determined.

At present, more attention has been paid to the role of PVN nNOS than to eNOS or iNOS in sympathetic modulation [20,22,26,27]. To demonstrate the roles of PVN nNOS, eNOS and iNOS in the regulation of sympathetic tone, we applied selective inhibitors of nNOS, eNOS and iNOS into PVN. The identical inhibitory phenomenon observed for L-NAME was observed for nNOS inhibitor (N-Propyl) and eNOS inhibitor (L-NIO) microinjection into the PVN in IR rats. However, regarding iNOS inhibition using an iNOS inhibitor (SMT), we did not observe any significant change in basal SNA in the Control or IR rats. These findings suggest that nNOS and eNOS, but not iNOS, are involved in sympathetic modulation in the PVN but that their abilities to inhibit sympathetic activation were weakened in the IR state. In addition, we detected significant decreases in nNOS and eNOS protein expression in the PVN of IR rats, whereas no significant change in iNOS protein expression was observed between the IR and Control rats. Moreover, lower nNOS and eNOS protein levels were accompanied by corresponding changes in nNOS and eNOS activity (the dimeric/monomeric expression ratio of nNOS and eNOS was examined as an indicator of their activity). Therefore, the decrease in nNOS and eNOS protein expression and activity may be a major cause of the reduction in NO content in the PVN of IR rats.

The NOSs are homodimeric enzymes that generate NO, but under certain conditions, nNOS and eNOS may dissociate into their monomeric form, which causes the production of superoxide [44,45]. We recently found that superoxide anions in the PVN can cause sympathetic activation and they can react with and inactivate NO in the PVN, thereby regulating SNA, in obese rats (data not published). Furthermore, the excessive superoxide anion levels in the PVN participated in enhanced SNA in IR rats [34]. Thus, it is possible that the reduced generation of NO in the PVN partly promotes superoxide anion-induced sympathoexcitation during IR. Based on this study, it can be postulated that decreased protein expression and low activity levels of nNOS and eNOS in the PVN result in decreased NO content, which further facilitates the elevation of SNA in IR rats. NO may act indirectly in the PVN by affecting the release of other neurotransmitters such as GABA and glutamic acid [24,25,46]. Therefore, it is possible that a reduction in the NO content diminishes neuronal activity, resulting in a decrease in the release of inhibitory neurotransmitters such as GABA in the PVN in the IR state. Alternatively, the responsiveness of neurons to NO may be reduced in the IR state. Therefore, these unresolved issues need to be investigated in future studies.

Many disease states related to sympathetic overdrive, such as hypertension, HF, diabetes and obesity, may be associated with altered NO system function in the central nervous system [14–17,29]. The present study demonstrated that an altered NO system in the PVN is involved in sympathetic activation in the IR state. The overexpression of the nNOS gene in the PVN significantly alleviated the elevation in SNA in HF model rats [20]. The genetic deletion of eNOS in diabetic mice resulted in advanced and progressive nephropathy [47]. Furthermore, the knockout of both the eNOS and nNOS genes in mice caused spontaneous IR based on hyperinsulinemic-euglycemic clamp studies [48]. These findings indicated that nNOS and eNOS in the PVN may represent potent targets for inhibiting sympathetic activation during IR.

In summary, the present study provides evidence indicating that a specific central malfunction (the NO system in the PVN) results in altered SNA during IR. The reduction of NO generation in the PVN caused weakened sympathoinhibition in IR rats, and this alteration may contribute to the elevation of SNA observed in metabolic syndrome, diabetes mellitus and obesity. Therefore, our findings support the possibility of an intervention such as scavenging superoxide with tempol (superoxide dismutase mimetic), then gene transfer of nNOS or eNOS for increasing NO content to inhibit sympathetic activation and prevent the progression of complications related to the diseases mentioned above.

## References

[1] Straznicky NE, Grima MT, Sari CI, Eikelis N, Lambert GW, Nestel PJ, et al A randomised controlled trial of the effects of pioglitazone treatment on sympathetic nervous system activity and cardiovascular function in obese subjects with metabolic syndrome. J Clin Endocrinol Metab. 2014;99: E1701–E1707. 10.1210/jc.2014-1976 24937541

[2] Iyngkaran P, Anavekar N, Majoni W, Thomas MC. The role and management of sympathetic overactivity in cardiovascular and renal complications of diabetes. Diabetes Metab. 2013;39: 290–298. 10.1016/j.diabet.2013.05.002 23871308

[3] Cozzolino D, Esposito K, Palmiero G, De Bellis A, Furlan R, Perrotta S, et al Cardiac autonomic regulation in response to a mixed meal is impaired in obese children and adolescents: the role played by insulin resistance. J Clin Endocrinol Metab. 2014;99: 3199–3207. 10.1210/jc.2013-4211 24840808

[4] Dong H, Jiang X, Liang T, Zou Y, Guan T, Peng M, et al Transradial renal denervation for the treatment of resistant hypertension. J Invasive Cardiol. 2014;26: 322–327. 24993989

[5] Grundy SM, Brewer HB Jr, Cleeman JI, Smith SC Jr, Lenfant C. Definition of Metabolic Syndrome:Report of the National Heart, Lung, and Blood Institute/American Heart Association Conference on Scientiic Issues Related to Definition. Circulation. 2004;109: 433–438. 14744958 10.1161/01.CIR.0000111245.75752.C6

[6] Grassi G. Role of the sympathetic nervous system in human hypertension. Journal of Hypertension. 1998;16: 1979–1987. 9886886 10.1097/00004872-199816121-00019

[7] Amerena J, Julius S. The role of the autonomic nervous system in hypertension. Hypertension Research. 1995;18: 99–110. 7584925 10.1291/hypres.18.99

[8] Després JP, Lamarche B, Mauriège P, Cantin B, Dagenais GR, Moorjani S, et al Hyperinsulinemia as an independent risk factor for ischemic heart disease. N Engl J Med. 1996;334: 952–957. 8596596 10.1056/NEJM199604113341504

[9] Reaven GM. Role of insulin resistance in human disease. Diabetes. 1988;37: 1595–1607. 3056758 10.2337/diab.37.12.1595

[10] Julius S, Valentini M, Palatini P. Overweight and hypertension: a 2-way street? Hypertension. 2000;35: 807–813. 10720599 10.1161/01.hyp.35.3.807

[11] Grundy SM. What is the contribution of obesity to the metabolic syndrome? Endocrinology and Metabolism Clinics of North America. 2004;33: 267–282. 15158519 10.1016/j.ecl.2004.03.001

[12] Anderson EA, Hoffman RP, Balon TW, Sinkey CA, Mark AL. Hyperinsulinemia produces both sympathetic neural activation and vasodilation in normal humans. J Clin Invest. 1991;87: 2246–2252. 2040704 10.1172/JCI115260 PMC296986

[13] Landsberg L. Obesity and the insulin resistance syndrome. Hypertension Research 1996;19: S51–S55. 9240765 10.1291/hypres.19.supplementi_s51

[14] Coote JH, Yang Z, Pyner S, Deering J. Control of sympathetic outflows by the hypothalamic paraventricular nucleus. Clin Exp Pharmacol Physiol. 1998;25: 461–463. 9673825 10.1111/j.1440-1681.1998.tb02235.x

[15] Zhang F, Sun HJ, Xiong XQ, Chen Q, Li YH, Kang YM, et al Apelin-13 and APJ in paraventricular nucleus contribute to hypertension via sympathetic activation and vasopressin release in SHR. Acta Physiol (Oxf). 2014; 212: 17–27. 24995933 10.1111/apha.12342

[16] Huang BS, Chen A, Ahmad M, Wang HW, Leenen FH. Mineralocorticoid and angiotensin II type 1 receptors in the paraventricular nucleus contribute to sympathetic hyperactivity and cardiac dysfunction in rats post myocardial infarction. J Physiol. 2014;592: 3273–3286. 10.1113/jphysiol.2014.276584 24951624 PMC4146375

[17] Xiong XQ, Chen WW, Han Y, Zhou YB, Zhang F, Gao XY, et al Enhanced adipose afferent reflex contributes to sympathetic activation in diet-induced obesity hypertension. Hypertension. 2012;60: 1280–1286. 10.1161/HYPERTENSIONAHA.112.198002 23033372

[18] Zhang L, Xiong XQ, Fan ZD, Gan XB, Gao XY, Zhu GQ. Involvement of enhanced cardiac sympathetic afferent reflex in sympathetic activation in early stage of diabetes. J Appl Physiol (1985). 2012;113: 47–55. 22582215 10.1152/japplphysiol.01228.2011

[19] Patel KP1, Li YF, Hirooka Y. Role of nitric oxide in central sympathetic outflow. Exp Biol Med (Maywood). 2001;226: 814–824. 11568303 10.1177/153537020122600902

[20] Zheng H, Liu X, Li Y, Sharma NM, Patel KP. Gene transfer of neuronal nitric oxide synthase to the paraventricular nucleus reduces the enhanced glutamatergic tone in rats with chronic heart failure. Hypertension. 2011;58: 966–973. 10.1161/HYPERTENSIONAHA.111.176222 21968757 PMC3212103

[21] Zhou YB, Sun HJ, Chen D, Liu TY, Han Y, Wang JJ, et al Intermedin in paraventricular nucleus attenuates sympathetic activity and blood pressure via nitric oxide in hypertensive rats. Hypertension. 2014;63: 330–337. 10.1161/HYPERTENSIONAHA.113.01681 24218431

[22] Sharma NM, Zheng H, Mehta PP, Li YF, Patel KP. Decreased nNOS in the PVN leads to increased sympathoexcitation in chronic heart failure: role for CAPON and Ang II. Cardiovasc Res. 2011;92: 348–357. 10.1093/cvr/cvr217 21831995 PMC3193834

[23] Xue B, Singh M, Guo F, Hay M, Johnson AK. Protective actions of estrogen on angiotensin II-induced hypertension: role of central nitric oxide. Am J Physiol Heart Circ Physiol. 2009;297: H1638–H1646. 10.1152/ajpheart.00502.2009 19734362 PMC2781378

[24] Busnardo C, Ferreira-Junior NC, Cruz JC, Machado BH, Correa FM, Resstel LB. Cardiovascular responses to ATP microinjected into the paraventricular nucleus are mediated by nitric oxide and NMDA glutamate receptors in awake rats. Exp Physiol. 2013;98: 1411–1421. 10.1113/expphysiol.2013.073619 23733521

[25] Martins-Pinge MC, Mueller PJ, Foley CM, Heesch CM, Hasser EM. Regulation of arterial pressure by the paraventricular nucleus in conscious rats: interactions among glutamate, GABA, and nitric oxide. Front Physiol. 2013;3: 490 10.3389/fphys.2012.00490 23316170 PMC3540931

[26] Zheng H, Liu X, Patel KP. Angiotensin-converting enzyme 2 overexpression improves central nitric oxide-mediated sympathetic outflow in chronic heart failure. Am J Physiol Heart Circ Physiol. 2011;301: H2402–H2412. 10.1152/ajpheart.00330.2011 21963832 PMC3737011

[27] Sharma NM, Llewellyn TL, Zheng H, Patel KP. Angiotensin II-mediated posttranslational modification of nNOS in the PVN of rats with CHF: role for PIN. Am J Physiol Heart Circ Physiol. 2013;305: H843–H55. 10.1152/ajpheart.00170.2013 23832698 PMC3761348

[28] Zheng H, Mayhan WG, Bidasee KR, Patel KP. Blunted nitric oxide-mediated inhibition of sympathetic nerve activity within the paraventricular nucleus in diabetic rats. Am J Physiol Regul Integr Comp Physiol. 2006;290: R992–R1002. 16322352 10.1152/ajpregu.00363.2005

[29] Tashiro Y, Yogo K, Serizawa K, Endo K. Nicorandil suppresses urinary protein excretion and activates eNOS in Dahl salt-sensitive hypertensive rats. Clin Exp Nephrol. 2014 [Epub ahead of print]. 10.1007/s10157-014-0998-6 24952900

[30] Gingerich S, Krukoff TL. Estrogen in the paraventricular nucleus attenuates L-glutamate-induced increases in mean arterial pressure through estrogen receptor beta and NO. Hypertension. 2006;48: 1130–1136. 17075034 10.1161/01.HYP.0000248754.67128.ff

[31] Yang WW, Krukoff TL. Nitric oxide regulates body temperature, neuronal activation and interleukin-1 beta gene expression in the hypothalamic paraventricular nucleus in response to immune stress. Neuropharmacology. 2000;39: 2075–2089. 10963751 10.1016/s0028-3908(00)00054-x

[32] Yamaguchi N, Ogawa S, Okada S. Cyclooxygenase and nitric oxide synthase in the presympathetic neurons in the paraventricular hypothalamic nucleus are involved in restraint stress-induced sympathetic activation in rats. Neuroscience. 2010;170: 773–781. 10.1016/j.neuroscience.2010.07.051 20678554

[33] Yamaguchi N, Okada S, Usui D, Yokotani K. Nitric oxide synthase isozymes in spinally projecting PVN neurons are involved in CRF-induced sympathetic activation. Auton Neurosci. 2009;148: 83–89. 10.1016/j.autneu.2009.02.009 19307158

[34] Sun HJ, Zhou H, Feng XM, Gao Q, Ding L, Tang CS, et al Superoxide anions in the paraventricular nucleus mediate cardiac sympathetic afferent reflex in insulin resistance rats. Acta Physiol (Oxf). 2014;212: 267–282. 25307720 10.1111/apha.12405

[35] Ding L, Tong N, Feng XM, Chen D, Wang HS, Wang Y, et al Adipose afferent reflex response to insulin is mediated by melanocortin 4 type receptors in the paraventricular nucleus in insulin resistance rats. Acta Physiol (Oxf). 2015 [Epub ahead of print]. 10.1111/apha.12502 25846948

[36] Bradford MM. A rapid and sensitive method for the quantitation of microgramquantities of protein utilizing the principle of protein-dye binding. Anal Biochem. 1976; 72: 248–254. 942051 10.1016/0003-2697(76)90527-3

[37] Klatt P, Schmidt K, Lehner D, Glatter O, Bachinger HP, Mayer B. Structural analysis of porcine brain nitric oxide synthase reveals a role for tetrahydrobiopterin and L-arginine in the formation of an SDS-resistant dimer. EMBO J. 1995;14: 3687–3695. 7543842 10.1002/j.1460-2075.1995.tb00038.x PMC394443

[38] Hwang IS, Ho H, Hoffman BB, Reaven GM. Fructose-induced insulin resistance and hypertension in rats. Hypertension. 1987;10: 512–516. 3311990 10.1161/01.hyp.10.5.512

[39] Catena C, Giacchetti G, Novello M, Colussi G, Cavarape A, Sechi LA. Cellular mechanisms of insulin resistance in rats with fructose-induced hypertension. Am J Hypertens. 2003;16: 973–978. 14573337 10.1016/s0895-7061(03)01002-1

[40] Iyer SN, Katovich MJ. Effect of chronic losartanpotassium treatment on fructose-induced hypertension. Life Sci. 1994;55: PL139–PL144. 8041226 10.1016/0024-3205(94)00750-0

[41] Grassi G. Sympathetic overdrive and cardiovascular risk in the metabolic syndrome. Hypertens Res. 2006;29: 839–847. 17345783 10.1291/hypres.29.839

[42] Krukoff TL. Central regulation of autonomic function: No brakes? Clin Exp Pharmacol Physiol. 1998;25: 474–478. 9673828 10.1111/j.1440-1681.1998.tb02238.x

[43] Zhang K, Mayhan WG, Patel KP. Nitric oxide within the paraventricular nucleus mediates changes in renal sympathetic nerve activity. Am J Physiol Regul Integr Comp Physiol. 1997;273: R864–R872. 10.1152/ajpregu.1997.273.3.R864 9321861

[44] Riethmüller C, Gorren AC, Pitters E, Hemmens B, Habisch HJ, Heales SJ, et al Activation of neuronal nitric-oxide synthase by the 5-methyl analog of tetrahydrobiopterin. Functional evidence against reductive oxygen activation by the pterin cofactor. J Biol Chem. 1999;274: 16047–16051. 10347155 10.1074/jbc.274.23.16047

[45] Cortes-González C, Barrera-Chimal J, Ibarra-Sánchez M, Gilbert M, Gamba G, Zentella A, et al Opposite effect of Hsp90α and Hsp90β on eNOS ability to produce nitric oxide or superoxideanion in human embryonic kidney cells. Cell Physiol Biochem. 2010;26: 657–668. 10.1159/000322333 21063103

[46] Togashi H, Sakuma I, Yoshioka M, Kobayashi T, Yasuda H, Kitabatake A, et al A central nervous system action of nitric oxide in blood pressure regulation. J Pharmacol Exp Ther. 1992;262: 343–347. 1378094

[47] Takahashi T, Harris RC. Role of Endothelial Nitric Oxide Synthase in Diabetic Nephropathy: Lessons from Diabetic eNOS Knockout Mice. J Diabetes Res. 2014;2014: 590541 10.1155/2014/590541 25371905 PMC4211249

[48] Shankar RR, Wu Y, Shen HQ, Zhu JS, Baron AD. Mice with gene disruption of both endothelial and neuronal nitric oxide synthase exhibit insulin resistance. Diabetes. 2000;49: 684–687. 10905473 10.2337/diabetes.49.5.684

